# CD276 is a promising biomarker for the prognosis of clear cell renal cell carcinoma

**DOI:** 10.1002/kjm2.12891

**Published:** 2024-08-29

**Authors:** Yan‐Hang Yu, Jian‐Hao Xu, Hao Chen, Yu‐Xin Lin, Jun Ou‐Yang, Zhi‐Yu Zhang

**Affiliations:** ^1^ Department of Urology The First Affiliated Hospital of Soochow University Suzhou China; ^2^ Department of Pathology The First People's Hospital of Kunshan Suzhou China

**Keywords:** CD276, clear cell renal cell carcinoma, nomogram, progression‐free interval, tumor diameter

## Abstract

This study aimed to investigate the role of cluster of differentiation 276 (CD276) in evaluating the prognosis of clear cell renal carcinoma (ccRCC) and to build a nomogram for predicting ccRCC progression post‐surgery. Using data downloaded from The Cancer Genome Atlas (TCGA) database, we constructed a Kaplan–Meier (KM) curve depicting the relationship between CD276 expression levels and the progression‐free interval (PFI) in 539 ccRCC cases. We further validated this by plotting a KM curve of the relationship between CD276 expression levels and PFI in 116 ccRCC patients from our hospital. Using clinical data collected from 116 patients, we identified independent risk factors affecting postoperative PFI in patients with ccRCC through univariate and multivariate COX analyses and created a nomogram for visual representation. Both TCGA and clinical data revealed a negative correlation between the expression levels of CD276 and PFI (*p* < 0.05). Univariate COX analysis revealed that the prognostic nutritional index, neutrophil‐to‐lymphocyte ratio, platelet‐to‐lymphocyte ratio, systemic inflammatory index, World Health Organization grading, tumor diameter, CD276 expression levels, T stage, and N stage were related to PFI (*p* < 0.05). Furthermore, multivariate COX analysis indicated that tumor diameter and CD276 expression levels were independent risk factors for postoperative PFI in patients with ccRCC (*p* < 0.05). The calibration curve of the established nomogram exhibited a slope close to 1, with a Hosmer–Lemeshow goodness‐of‐fit test result of 2.335 and a *p*‐value of 0.311. In patients with ccRCC, a negative correlation was noted between tumor CD276 expression and PFI. The larger the tumor diameter and the higher the tumor CD276 expression level, the shorter is the PFI.

## 1 INTRODUCTION

Renal cell carcinoma (RCC) rank among the most common cancers globally, particularly affecting men and the elderly.^1^ The most common subtype, clear cell renal cell carcinoma (ccRCC), represents 70%–80% share of all RCC cases in adults.^2^ Surgical resection is the primary treatment for ccRCC,^3^ but 40% of patients experience recurrence or progression post‐surgery.^4^ Advanced or metastatic ccRCC is commonly treated with molecular‐targeted drugs like Sunitinib and Everolimus as the first‐ and second‐line treatment modalities.^5^ However, these treatments typically extend the progression‐free interval (PFI) by no more than 12 months.^6^, ^7^ Therefore, there is an urgent need to identify new molecular targets affecting ccRCC patients with ccRCC. This could serve as a reference for patient prognosis prediction and provide targets for the development of new targeted drugs, consequently enhancing beneficial PFI in patients.

The B7‐Homologue 3 molecule (B7‐H3), also identified as cluster of differentiation 276 (CD276), was first introduced as a member of the B7/CD28 immune co‐stimulatory family, as revealed by Chapoval et al.^8^ in 2001. Recently, CD276 had been found to be overexpressed in various cancers, including breast, non‐small cell lung, and ovarian cancer,^9^, ^10^, ^11^, ^12^ suggesting its potential as a promising immunotherapeutic target. Our previous studies confirmed that the expression of CD276 is higher in ccRCC tumor tissues than in normal adjacent tissues, indicating its role as a molecular marker for diagnosing ccRCC.^13^, ^14^ However, the relationship between CD276 expression and ccRCC prognosis has not been well documented. Therefore, we aimed to further validate the relationship between the expression level of CD276 and ccRCC prognosis. This could provide a clinical reference for prognosis prediction in patients with ccRCC after surgical treatment and offer documentary support for adopting CD276 as a molecular target for ccRCC treatment in subsequent studies.

## 2 MATERIALS AND METHODS

This study aligns with the established standards of the STARD guidelines.^15^

### 2.1 TCGA data description

Gene expression data from 613 patients were procured from the Kidney Renal Clear Cell Carcinoma (KIRC) project in The Cancer Genome Atlas (TCGA) database (https://portal.gdc.cancer.gov). A retrospective analysis was conducted on 539 cases with PFI data. Data were processed using spliced transcript alignment to a reference (STAR) pipeline and extracted in the transcripts per million (TPM) format. The dataset proposes estimates of messenger RNA (mRNA) transcription at the level of log2(value+1)‐transformed RNA‐Seq by expectation–maximization normalized counts. For statistical evaluations, we used false discovery rate (FDR) rate‐corrected *q*‐values adjusted to 0.05. We used COX regression to examine the relationship between CD276 expression levels and PFI in patients with ccRCC and depicted this association using a Kaplan–Meier (KM) curve.

### 2.2 Clinical data description

This retrospective study included 116 patients who underwent surgery and were pathologically diagnosed with ccRCC at the First Affiliated Hospital of Soochow University between January 2016 and December 2018. The inclusion and exclusion criteria were: (1) patients who had received chemotherapy, radiotherapy, immunotherapy, or other treatments prior to surgery were excluded; (2) patients with other malignant tumors or infectious diseases such as AIDS, were excluded; (3) patients whose original pathological tissue sections were not preserved by our institute were excluded; and (4) patients with complete clinical data and willingness to undergo follow‐up investigations were included. The collected clinical data included sex, age, body mass index (BMI), comorbid high blood pressure (HBP), comorbid diabetes mellitus (DM), prognostic nutritional index (PNI), neutrophil‐to‐lymphocyte ratio (NLR), platelet‐to‐lymphocyte ratio (PLR), lymphomonocyte ratio (LMR), systemic immunoinflammatory index (SII), World Health Organization (WHO) grading system, tumor diameter, surgical method, expression levels of CD276, PFI time, T stage, and N stage. All blood samples were collected from peripheral blood prior to surgery. Based on whether there was postoperative progression or metastasis, all patients were divided into recurrence and non‐recurrence groups for further analysis. This study was approved by the Ethics Committee of the First Affiliated Hospital of Soochow University (Approval No. 231, 2024). All participants provided written informed consent before participating in the study. All patient information was anonymized for privacy.

### 2.3 Specimen collection

Renal tumor specimens were collected from the pathology department of our hospital. These specimens were stored at −80˚C to ensure proper preservation for subsequent experiments.

### 2.4 Quantitative real‐time polymerase chain reaction

Following the manufacturer's instructions, total ribonucleic acid (RNA) with TRIzol reagent (Takara, Dalian, China). A reverse transcription kit (Takara, Dalian, China) was used to synthesize complementary deoxyribonucleic acid (cDNA). The synthesized cDNA was, then, mixed with 12.5 μL of 2× SYBR Green Master Mix (Roche Diagnostics GmbH, Mannheim, Germany), 1 μL of 400 nM primers, and 9.5 μL of RNase‐free water for qPCR. The primer sequences used for rt‐PCR were introduced in our previous study.^14^ Specifically, they are as follows: GAPDH‐F: GATGCCCCCATGTTCGTCAT; GAPDH‐R: TCTTCTGGGTGGCAGTGATG; CD276‐F: AGGGCAGCCTATGACATTCC; CD276‐R: TGTCTTGGAGCCTTCTCCCT. An Applied Biosystems 7500 Fast Dx Real‐Time PCR Instrument (Thermo Fisher Scientific, USA) was used to perform RT‐PCR with SYBR Premix Ex Taq II (Roche Diagnostics GmbH, Mannheim, Germany). We calculated the relative gene expression using the ΔΔCT method, as reported in our prior study.^16^

### 2.5 Follow‐up

The cut‐off date for follow‐up was December 31, 2023. The PFI is defined as the period between the day of surgery and either the set deadline or the moment at which progression or recurrence is detected.^17^ Patients categorized as low‐grade (grades I and II) in the WHO grading system are monitored using a scheme that includes check‐ups every 6 weeks post‐surgery, biannually for 3 years post‐surgery, and yearly thereafter. In contrast, patients with high‐grade disease (grades III and IV) under the same WHO grading system have different follow‐up schedules. Monitoring starts with visits every 6 weeks after surgery, quarterly up to the second year, biannually during the third year, and annually thereafter. Follow‐up sessions were conducted via phone calls and outpatient consultations. Each follow‐up visit requires a physical examination and a repeat of blood tests, including complete blood count and biochemical profile. Chest, abdominal, and pelvic computerized tomography scans are performed every other follow‐up visit, with at least one scan conducted annually.

### 2.6 Statistical analysis

We had recourse to R software (version 4.2.1) for all data analyses, leveraging various essential packages including “stats [4.2.1], survival [3.3.1], survminer [0.4.9], ggplot2 [3.3.6], and rms [6.3‐0]”. To compare qualitative data between the two groups, we applied a *t*‐test or Wilcoxon test, as necessary. Cumulative data comparisons were performed using the *χ* ^2^ test. Survival analysis was based on the log‐rank test and KM curve. Univariate and multivariate COX regression analyses were used to identify independent risk factors. We constructed a nomogram for predicting PFI, the accuracy of which was assessed using a calibration curve. The results were considered statistically significant at *p* < 0.05. The necessity for at least 103 individuals to participate was determined based on sample size calculations conducted using R software.^18^ Finally, to ensure integrity and accuracy, two senior statisticians independently verified all the data.

## 3 RESULTS

### 3.1 Expression level of CD276 is inversely correlated with the PFI of patients with ccRCC in TCGA dataset

**FIGURE 1:**
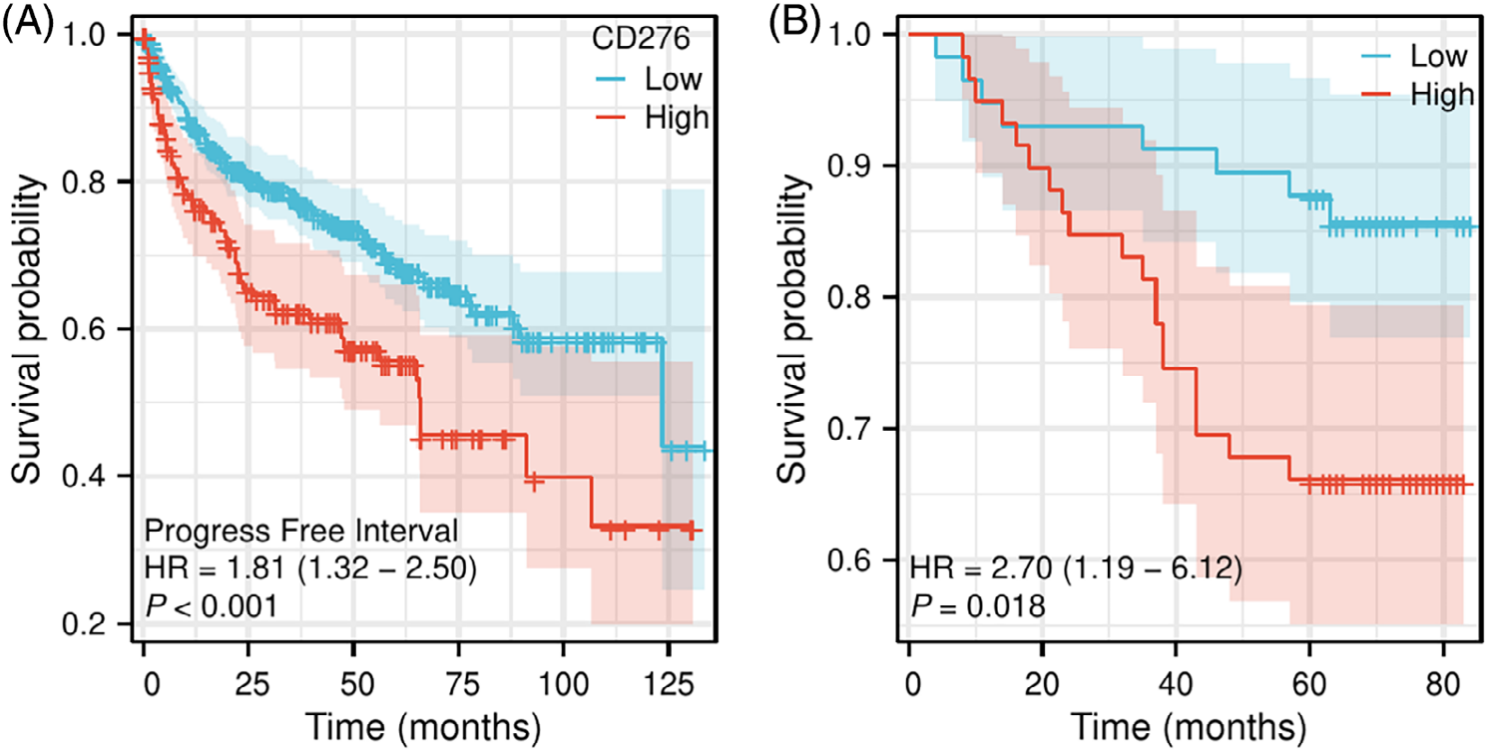
Survival curve of PFI for patients with high or low CD276 expression in ccRCC. (A) Survival curve of PFI for patients with high or low CD276 expression in ccRCC in TCGA dataset. (B) Survival curve of PFI for patients with high or low CD276 expression in ccRCC in clinical dataset. ccRCC, clear cell renal cell carcinoma; CD276, cluster of differentiation 276; HR, hazard ratio; PFI, progression‐free interval; TCGA, The Cancer Genome Atlas.

Survival data from 539 patients downloaded from the TCGA‐KIRC database were used to study the relationship between the expression level of CD276 and PFI. The resultant Kaplan–Meier curve revealed that patients with high CD276 expression had worse PFI (hazard ratio (HR) = 1.81, 95% confidence interval (CI) 1.266–2.592, *p* = 0.0002) (Figure 1A).

### 3.2 Comparison of the differences in clinicopathological characteristics between the recurrence and non‐recurrence group in clinical dataset

In total, 116 patients with ccRCC who underwent surgical treatment at our hospital were included in this study. All patients were divided into the recurrence and non‐recurrence groups based on whether they experienced disease recurrence or progression. The recurrence and non‐recurrence groups included 28 and 88 patients, respectively. Statistically significant differences were found between the two groups in terms of PNI, NLR, PLR, SII, WHO grading, tumor diameter, surgical method, CD276 expression, T stage, and N stage (*p* < 0.05) (Table 1).

**TABLE 1:** Comparison of clinicopathological characteristics between the recurrence group and the non‐recurrence group.

Characteristics	Recurrence group	Non‐recurrence group	*p* value
*n*	28	88	
Gender, *n* (%)			0.588
Female	8 (28.6%)	30 (34.1%)	
Male	20 (71.4%)	58 (65.9%)	
Age (year), median (IQR)	60 (50, 66.25)	60 (51, 69)	0.588
BMI (kg/m^2^), mean ± SD	24.413 ± 2.5727	24.608 ± 2.8141	0.746
HBP, *n* (%)			0.133
No	15 (53.6%)	33 (37.5%)	
Yes	13 (46.4%)	55 (62.5%)	
DM, *n* (%)			0.467
No	22 (78.6%)	63 (71.6%)	
Yes	6 (21.4%)	25 (28.4%)	
PNI (%), median (IQR)	47.375 (44.65, 50.125)	50.5 (46.987, 52.688)	0.003
NLR, median (IQR)	2.265 (2.055, 2.5725)	1.985 (1.7125, 2.37)	0.015
PLR, median (IQR)	133.33 (117.92, 196.99)	114.48 (98.532, 148.35)	0.025
LMR, median (IQR)	3.765 (3.2125, 4.12)	3.925 (3.3, 4.7125)	0.141
SII, median (IQR)	441.76 (382.79, 600.7)	422.73 (314.33, 512.49)	0.086
WHO grading, *n* (%)			<0.001
High grade	14 (50%)	15 (17%)	
Low grade	14 (50%)	73 (83%)	
Diameter (mm), median (IQR)	66 (50, 80)	40 (30, 53.5)	<0.001
Surgery, *n* (%)			0.266
Radical nephrectomy	18 (64.3%)	46 (52.3%)	
Partial nephrectomy	10 (35.7%)	42 (47.7%)	
CD276, median (IQR)	1.3719 (0.77687, 1.6875)	0.7542 (0.42488, 1.0675)	<0.001
T stage, *n* (%)			<0.001
T1a	4 (14.3%)	49 (55.7%)	
T1b	12 (42.9%)	37 (42%)	
T2a	9 (32.1%)	1 (1.1%)	
T2b	3 (10.7%)	1 (1.1%)	
N stage, *n* (%)			0.014
N0	24 (85.7%)	87 (98.9%)	
N1	4 (14.3%)	1 (1.1%)	

*Abbreviations*: BMI, body mass index; CD276, cluster of differentiation 276; DM, diabetes mellitus; HBP, high blood pressure; IQR, interquartile range; LMR, lymphomonocyte ratio; NLR, neutrophil‐to‐lymphocyte ratio; PLR, platelet‐to‐lymphocyte ratio; PNI, prognostic nutritional index; SD, standard deviation; SII, systemic immunoinflammatory index; WHO, World Health Organization.

### 3.3 Univariate and multivariate COX regression analyses of influencing factors on PFI in clinical dataset

The results of the COX univariate analysis indicate that PNI, NLR, PLR, SII, WHO grade, tumor diameter, CD276 expression levels, T stage, and N stage were correlated with PFI (*p* < 0.05) (Table 2). Furthermore, COX multivariate analysis revealed that tumor diameter and CD276 expression levels were independent risk factors influencing PFI (*p* < 0.05) (Table 2).

**TABLE 2:** Univariate and multivariate COX regression analyses of influencing factors on PFI.

Characteristics	Total (*N*)	Univariate analysis		Multivariate analysis	
		Hazard ratio (95% CI)	*p* value	Hazard ratio (95% CI)	*p* value
Gender	116				
Female	38	Reference			
Male	78	1.333 (0.587–3.028)	0.492		
Age (year)	116	0.992 (0.960–1.026)	0.653		
BMI (kg/m^2^)	116	0.976 (0.853–1.116)	0.721		
HBP	116				
No	48	Reference			
Yes	68	0.564 (0.269–1.186)	0.131		
DM	116				
No	85	Reference			
Yes	31	0.734 (0.298–1.812)	0.503		
PNI (%)	116	0.856 (0.776–0.943)	0.002	0.969 (0.868–1.080)	0.568
NLR	116	2.197 (1.334–3.618)	0.002	2.328 (0.893–6.072)	0.084
PLR	116	1.009 (1.002–1.017)	0.016	1.000 (0.983–1.017)	0.972
LMR	116	0.735 (0.508–1.063)	0.102		
SII	116	1.002 (1.001–1.003)	<0.001	0.999 (0.995–1.002)	0.501
WHO grading	116				
High grade	29	Reference		Reference	
Low grade	87	0.271 (0.129–0.569)	<0.001	0.921 (0.300–2.826)	0.885
Diameter (mm)	116	1.029 (1.018–1.040)	<0.001	1.010 (1.006–1.064)	0.049
Surgery	116				
Radical nephrectomy	64	Reference			
Partial nephrectomy	52	0.674 (0.311–1.461)	0.318		
CD276	116	5.202 (2.252–12.017)	<0.001	3.250 (1.060–10.500)	0.037
T stage	116				
T1a	53	Reference		Reference	
T1b	49	3.602 (1.161–11.173)	0.026	2.538 (0.432–14.911)	0.303
T2a	10	31.641 (9.574–104.567)	<0.001	7.717 (0.274–217.201)	0.230
T2b	4	12.092 (2.698–54.189)	0.001	5.198 (0.039–688.062)	0.508
N stage	116				
N0	111	Reference		Reference	
N1	5	6.159 (2.111–17.967)	<0.001	1.542 (0.329–7.217)	0.582

*Abbreviations*: BMI, body mass index; CD276, cluster of differentiation 276; CI, confidence interval; DM, diabetes mellitus; HBP, high blood pressure; LMR, lymphocyte ratio; NLR, neutrophil‐to‐lymphocyte ratio; PLR, platelet‐to‐lymphocyte ratio; PNI, prognostic nutritional index; SII, systemic inflammatory index; WHO, World Health Organization.

### 3.4 Plotting the KM curve for PFI of patients with ccRCC in relation to CD276 expression level in clinical dataset

The follow‐up time for patients in this study ranged from 4 to 84 months, with a median of 66 months and an interquartile range (IQR) of 60.25–70 months. Based on the median expression level of CD276, patients were divided into two groups: a high and low expression group. The KM curve indicated that patients in the low CD276 expression group had a longer PFI than those in the high expression group (Figure 1B). A Log‐rank test revealed that these differences were statistically significant (HR = 2.7, 95% CI = 1.276–5.618, *p* = 0.0138).

### 3.5 Construction of a postoperative PFI prediction model for patients with ccRCC in clinical dataset

**FIGURE 2:**
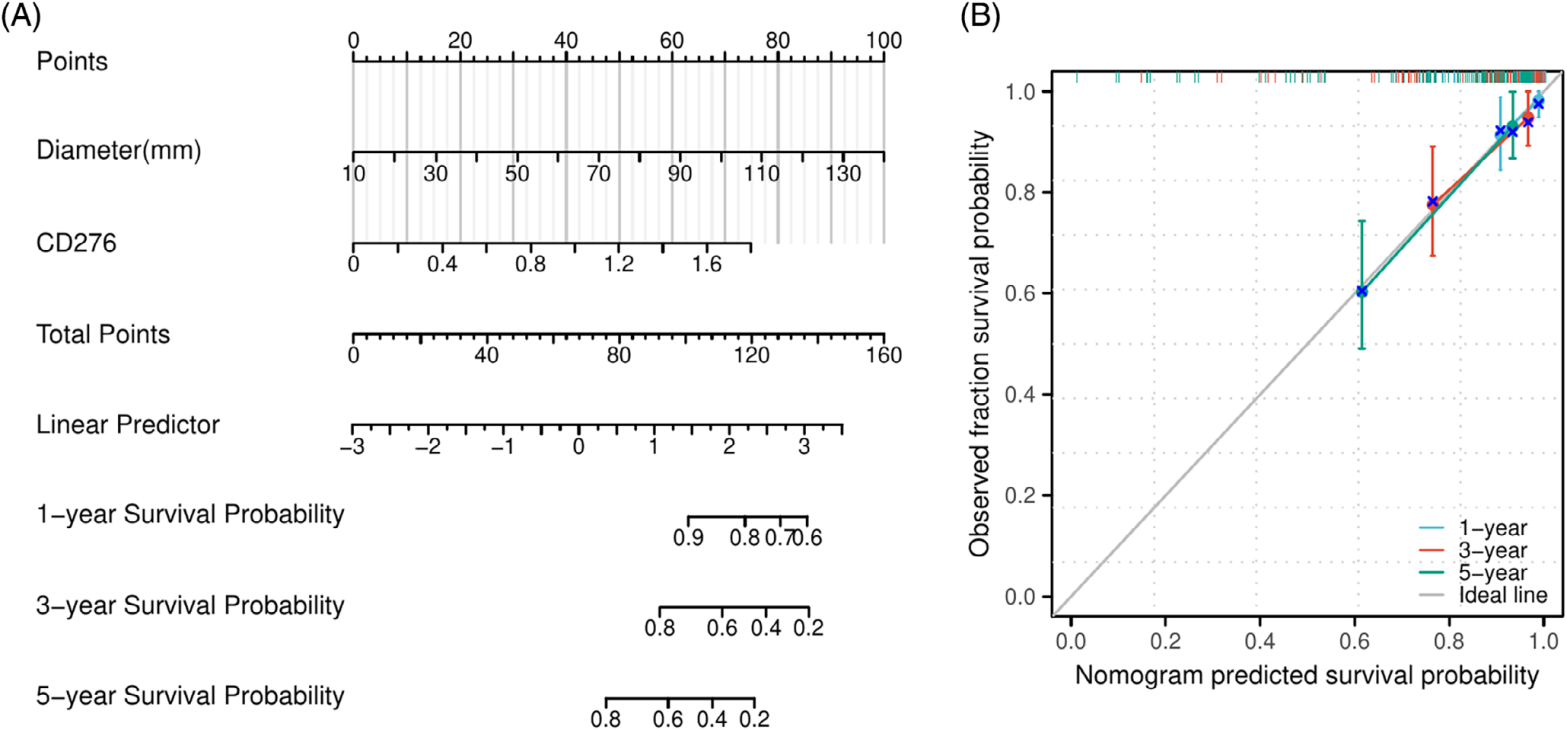
Establishment of a nomogram for predicting postoperative PFI in patients with ccRCC. (A) A nomogram for predicting 1‐, 3‐, and 5‐year PFI in patients with ccRCC. (B) Calibration of the nomogram model for PFI. ccRCC, clear cell renal cell carcinoma; CD276, cluster of differentiation 276; PFI, progression‐free interval.

Based on the results of the *multivariate* COX regression analysis, a nomogram for postoperative PFI prediction in patients with ccRCC was created (c‐index 0.795, 95% CI, 0.756–0.835) (Figure 2A). The calibration curve demonstrated a slope close to 1, with a Hosmer–Lemeshow goodness‐of‐fit test value of 2.335 and a *p* value of 0.311 (Figure 2B).

## 4 DISCUSSION

RCC, the predominant form of urogenital cancer, has a fatality percentage spanning between 30% and 40%.^19^ ccRCC is the predominant histological and molecular subtype of RCC and often results from mutations in VHL gene.^20^ Approaches, such as nephron‐sparing surgery aim to preserve kidney function in patients, whereas radical and partial nephrectomies are used to remove smaller tumors.^21^, ^22^ Although surgery is a viable treatment for RCC, distant metastases occur in 20%–30% of patients, and 2%–5% of patients experience local recurrence.^23^ Molecular targeted therapy is the preferred treatment option for patients with advanced or metastatic ccRCC, primarily targeting pathways, such as vascular endothelial growth factor (VEGF), platelet‐derived growth factor receptor (PDGFR), and mechanistic target of rapamycin (mTOR).^19^ However, the molecular‐targeted drugs currently used in the clinic have a limited impact on patient PFI, which, on average, does not exceed 1 year.^6^, ^7^ Therefore, the discovery and clinical studies of new molecular targets that can affect the prognosis of patients with ccRCC are urgently needed.

CD276 is a comparatively recent addition to the B7/CD28 family of immune costimulatory molecules^8^ and has been found to be highly expressed in various forms of cancer.^9^, ^10^, ^11^, ^12^ Recent publications have reported that CD276 is more abundantly expressed in ccRCC tumor tissues than in adjacent normal tissues, suggesting that CD276 could be used as a biological marker for predicting ccRCC.^24^, ^25^ Our previous studies also confirmed that CD276 is highly expressed in ccRCC tissues; however, its expression is relatively low in tissues surrounding the tumor.^13^, ^14^ However, only a few studies have investigated the relationship between CD276 expression and postoperative PFI in patients with ccRCC. To our knowledge, this is the first study to specifically emphasize on the relationship between CD276 expression and postoperative PFI in patients with ccRCC. We conducted a long‐term follow‐up of 116 patients with ccRCC who underwent surgical treatment, with a recurrence or progression rate of 24.14% (28/116). Utilizing data from the TCGA database and our hospital's clinical data, we plotted KM curves and found that high CD276 expression corresponded to a shorter PFI. Thus, there was a negative correlation between CD276 expression and postoperative PFI in patients with ccRCC. This suggests that CD276 can serve not only as a biomarker for diagnosing ccRCC but also as a predictor of disease prognosis.

PNI is assessed based on the levels of serum albumin and lymphocyte counts in the peripheral blood.^26^ The PNI serves as an indicator of both nutritional and immunological conditions in patients with cancer and is regarded as a significant predictor of various solid cancer types including RCC.^27^ In accordance with our research, we found that the PNI was closely linked to postoperative recurrence or progression in patients with ccRCC. Neutrophils are crucial inflammatory cells of the human body. Under the influence of the tumor microenvironment, these cells possess the ability to promote tumor cell proliferation and the formation of new tumor blood vessels, ultimately accelerating tumor progression.^28^ Lymphocytes are considered the primary executors of the immune response in the body. In patients with tumors, due to the high metabolic nutritional consumption caused by the tumor tissue inside the body, a progressive decline in the host's immunity occurs. This results in a relative reduction in lymphocytes in cancer patients.^29^ Platelets primarily promote coagulation. However, numerous angiogenesis‐regulating proteins present within platelets can be exploited by tumor and tumor stromal cells. This process promotes the formation of new blood vessels, subsequently accelerating the proliferation and invasion of tumor cells.^30^ Inflammatory indicators derived from these parameters, such as the NLR, PLR, LMR, and SII, are significantly related to the diagnosis and prognosis of RCC.^29^, ^30^, ^31^, ^32^ Our study also revealed a correlation between NLR, PLR, SII, and postoperative PFI in patients with ccRCC. Additionally, NLR, PLR, and SII were directly correlated with early recurrence or metastasis in patients with postoperative ccRCC.

With an increase in the WHO pathological grading, the malignancy of the tumor also escalates, leading to a poorer prognosis in patients with ccRCC.^33^ In our research, we classified WHO pathological grades I and II as low‐grade and grades III and IV as high‐grade. The results showed that the PFI in the high‐grade group was significantly lower than that in the low‐grade group. In RCC, the tumor diameter is related to renal cancer staging. Furthermore, the larger the tumor diameter, the stronger its invasiveness and malignancy.^34^ Meanwhile, a study by Maxwell et al.^35^ found that with each 1 cm increase in the diameter of RCC tumors, the risk of tumor recurrence increased by 198%. Our study also confirmed that tumor diameter is an independent risk factor for recurrence or progression after surgery in patients with ccRCC. However, in our study, T stage showed differences between the recurrence and non‐recurrence groups in univariate analysis, but it did not emerge as an independent risk factor in multivariate analysis. In clinical practice, T stage is typically considered to be related to poorer prognosis.^36^ This discrepancy may arise because, although tumor diameter is correlated with T stage, tumor diameter is a continuous variable, whereas T stage is a categorical variable. Consequently, tumor diameter can more sensitively reflect the prognostic status of the tumor. Based on the results of COX multivariate analysis, we utilized the expression levels of CD276 and tumor diameter to construct a nomogram for predicting postoperative PFI in patients with ccRCC, which holds significant clinical application value.

Our study has certain limitations. First, this was a single‐center, small‐sample, retrospective study, and the conclusions drawn need to be further validated by large‐sample, multicenter, and prospective studies. Secondly, RCC has subtypes other than ccRCC. Owing to the limited data for other subtypes in our center, they were not included in our study, and further research on these subtypes is required to validate our results. Third, our study focused only on the correlation between CD276 expression levels and postoperative PFI in ccRCC without delving into the underlying mechanisms. Further studies are required to elucidate these mechanisms.

In conclusion, the expression level of CD276 is related to PFI after ccRCC surgery, with larger tumors correlating with higher levels of CD276 expression and thus shorter PFI. Thus, CD276 holds promise as a potential biomarker for ccRCC prognosis.

## CONFLICT OF INTEREST STATEMENT

All authors declare no conflict of interest.

## Data Availability

All original raw data of this study can be accessed from https://figshare.com/s/a4e2aa53d61d7c175742. Publicly available data were also obtained from TCGA database (KIRC, https://portal.gdc.cancer.gov/).
